# Resolving the bouba-kiki effect enigma by rooting iconic sound symbolism in physical properties of round and spiky objects

**DOI:** 10.1038/s41598-022-23623-w

**Published:** 2022-11-10

**Authors:** Mathilde Fort, Jean-Luc Schwartz

**Affiliations:** 1 Laboratoire de Psychologie Et NeuroCognition, UMR 5105, Université Grenoble Alpes, Grenoble, France; 2 Centre de Recherche en NeuroSciences de Lyon, UMR 5292, Université Lyon 1, Lyon, France; 3 Grenoble Images Parole Signal Automatique, UMR 5216, Université Grenoble Alpes, CNRS, Grenoble INP, Grenoble, France

**Keywords:** Human behaviour, Psychology

## Abstract

The “bouba-kiki effect”, where “bouba” is perceived round and “kiki” spiky, remains a puzzling enigma. We solve it by combining mathematical findings largely unknown in the field, with computational models and novel experimental evidence. We reveal that this effect relies on two acoustic cues: spectral balance and temporal continuity. We demonstrate that it is not speech-specific but rather rooted in physical properties of objects, creating audiovisual regularities in the environment. Round items are mathematically bound to produce, when hitting or rolling on a surface, lower-frequency spectra and more continuous sounds than same-size spiky objects. Finally, we show that adults are sensitive to such regularities. Hence, intuitive physics impacts language perception and possibly language acquisition and evolution too.

## Introduction

One central property of natural languages is the arbitrariness of the sign: the sounds of words do not generally inform about their meaning^1^. Still, systematic sound symbolic exceptions to this principle, found in a wide range of languages’ lexicons, have always intrigued scientists and non-scientists^2–4^. The so-called “bouba-kiki effect” or “maluma-takete effect” is the textbook case for sensitivity to iconic sound symbolism. When presented with a sound–shape matching task, perceivers almost systematically associate auditory pseudowords such as “bouba” or “maluma” with round or smooth shapes, and others, such as “kiki” or “takete,” with spiky or angular shapes^5,6^. Since its first report, the bouba-kiki effect has been robustly replicated across languages, cultures^7,8^ and stimuli, see^9,10^, for reviews, suggesting that it relies to some extent on universal cues^11,12^.

Yet, two major problems remain. First, the stimuli parameters involved in this effect are still unclear. For instance, both consonants and vowels appear to play a role in the bouba-kiki effect (though consonants to a larger extent^9^). Some studies suggest perceptual roundness could be induced by acoustic continuity^10,13^, phonetic continuancy^14^, spectral tilt^10^, pitch^13^, voice shimmer^10^, or body actions of the tongue^15^, lips^16^ or even hands^17^ that would be mirrored in visual round shapes. However, none of these parameters have yet shown to provide a coherent answer, accounting for the variety of experimental results in the literature. The second issue deals with the underlying mechanisms that could explain such audiovisual associations and whether the bouba-kiki effect is innate or can be learned through exposure to audiovisual regularities in the environment. In spite of being very robust in adults and shared by most language and cultures^11,12^, a meta-analysis^18^ showed that infants in their first year were only sensitive to a bouba effect (i.e. a tendency to associate bouba sounds with round shapes). The kiki effect (i.e. a tendency to associate kiki sounds with spiky shapes) was not present before the second year and remains smaller in size than the bouba effect, even in adults^9,11,12^. Thus, rather than being fully operational at birth, the bouba-kiki effect seems to increase with age, suggesting that it could be learned.

In this paper, we solve the first problem, by uncovering the universal acoustic parameters at play in this phenomenon. We then solve the second problem, by finding out the physical principles that could be at the basis of the cross-modal regularities underlying the bouba-kiki effect. We thus demonstrated how it could emerge through exposure to such regularities present in the environment. Finally, we discuss how this causal mechanistic account provides a complete and coherent resolution of the bouba-kiki effect enigma, and we raise some new perspectives on how language and human multimodal perception of natural scenes are intrinsically linked.

## Results

### Solving the first problem: a universal phenomenological model of the bouba-kiki effect

#### The sound of round is spectrally low and temporally smooth

Gathering published reports about the bouba-kiki effect in adults^9,10,13,14,16,19–26^, a consistent pattern emerges. Some speech sounds such as the nasal /m/, the voiced bilabial plosive /b/, the liquid /l/, or the back vowels /u o/ as in bouba and maluma are rather round (i.e. more often associated with round than spiky shapes). In contrast, others such as the unvoiced coronals /t k/ or the front vowels /i e/ as in kiki and takete are rather spiky (i.e. more often associated with spiky than round shapes). A phonetic analysis provides a straightforward interpretation of this phenomenology, showing that round speech sounds have lower-frequency spectra (or lower spectral balance) and display more continuous temporal fluctuations of their acoustic envelope (see Supplementary Information, for more details). In other words, *the sound of round is spectrally low and temporally smooth*. Surprisingly, while these simple acoustic properties have recently been mentioned for possibly being at the origin of the bouba-kiki effect^12^, they have never been tested.

#### Predicting the bouba-kiki effect from spectral balance and temporal continuity

In this section, we combined a meta-analysis with a computational modelling approach to quantitatively assess the possibility to predict the bouba-kiki effect from spectral balance and temporal continuity parameters. Thanks to their authors, we gathered data and speech stimuli from 8 publications (10 experiments, total of 394 participants, 1086 speech stimuli, five languages) on the bouba-kiki effect (see Table 2 in "Methods" for more details). In spite of the large dataset variability in terms of languages, linguistic contents and stimuli, we opted for a simple and single computational phenomenological model to extract spectral balance and temporal continuity parameters from the acoustic speech stimuli. The goal was to capture the trends shared by the entire dataset, even though it would likely be sub-optimal for each individual experiment.

**Figure 1:**
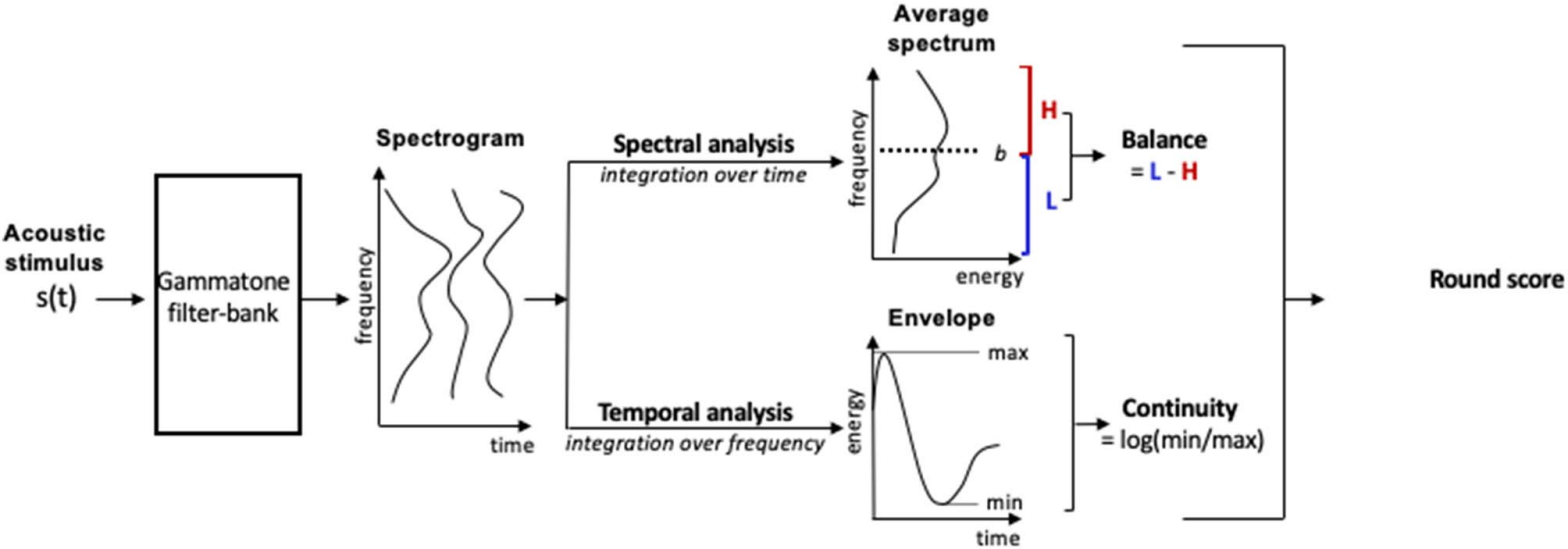
Schematic representation of the phenomenological model used for predicting round (over spiky) score based on the properties of each stimulus s (t) for each experiment. First, a spectrogram is generated thanks to a classical gammatone filter-bank inspired from auditory filtering^52^. Then, two independent analyses are applied. Spectral analysis (top pipeline) provides the Balance index defined as the difference between summed energy in low and high spectral components around a boundary *b* (Eq. 1). Temporal analysis (bottom pipeline) provides the Continuity index based on the ratio between the minimum and maximum energy through time within the corresponding stimulus (Eq. 2). Finally, the round (over spiky) score is linearly predicted from the combination of Balance and Continuity values. For more details, see "Methods" and Supplementary Information.

The phenomenological Balance x Continuity model is displayed in Fig. 1 and described in details in "Methods". For each speech stimulus, it performs basic acoustic analyses resulting in the computation of two parameters: the spectral balance between low and high frequencies (Balance parameter) and the temporal continuity between minimum and maximum amplitude (Continuity parameter). It then predicts a round (over spiky) score from these two parameters only.

**Figure 2:**
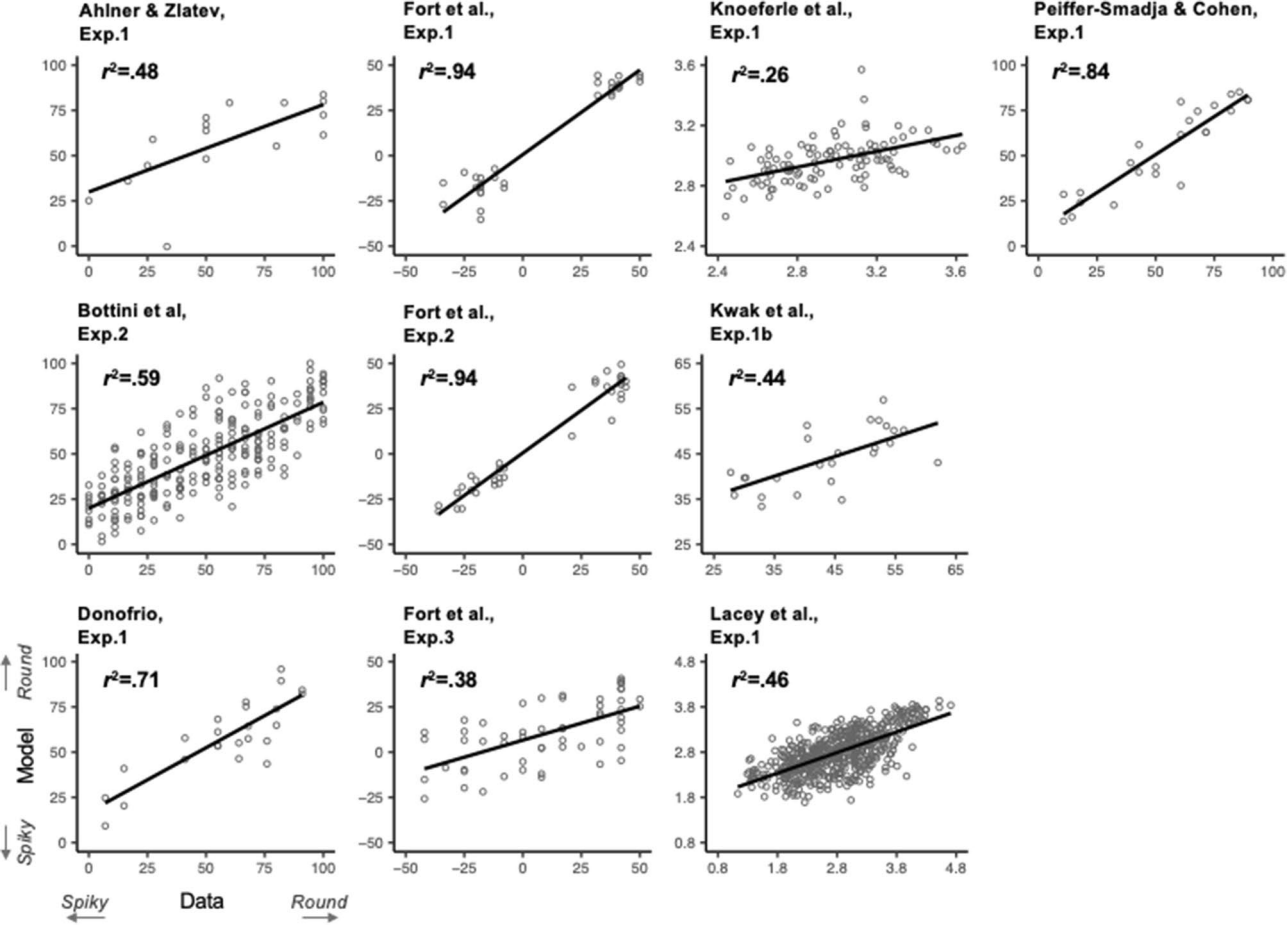
Model round score predictions (Model, y axis) as a function of Experimental round score data (Data, x axis, on the same scale as in the original studies, see Table 2 for more details) for the 10 experiments. For each speech stimulus (displayed by empty circles) in each experiment, the round score prediction in the model is obtained for the boundary *b* value providing the best fit for the corresponding experiment (cf. "Methods"). Linear regression lines and corresponding explained variance *r*^2^ between experimental and predicted round scores are added to each display.

To measure how well the Balance x Continuity model could predict the bouba-kiki effect, we conducted separate linear regressions for each experiment between the model’s round score predictions (Model) and the experimental round score (Data). For each experiment, we also computed experimental round score percentage of variance *r*^*2*^ explained by the model. These analyses were conducted using R^27^ and the work package “lm”. Their results are displayed in Fig. 2 and detailed in Table 1. All linear regressions were significant (alpha level: 0.05, see Table 1), explaining quite large percentages of variance (mean *r*^*2*^ = 60%, range: 26–94%, see Fig. 2). In other words, predictions of the Balance x Continuity model were very accurate for the entire bouba-kiki effect dataset (see also the Supplementary Information for more discussion).

**Table 1:** Statistical analysis of the linear regression fits for the 10 already published experiments and the Noise band experiment (synthetic non-speech stimuli) as well as their number of stimuli N.

Experiments	r^2^%	Standard error	t	p	N
Ahlner & Zlatev, 2010, Exp. 1	48	.13	3.62	0.003	16
Bottini et al. 2019, Exp. 2	59	.03	18.35	< 0.001	240
D’Onofrio, 2013, Exp. 1	71	.10	7.34	< 0.001	24
Fort et al. 2015, Exp.1	94	.05	20.82	< 0.001	32
Fort et al. 2015, Exp. 2	94	.04	23.18	< 0.001	36
Fort et al. Exp. 3	38	.07	5.70	< 0.001	56
Knoeferle et al. 2017, Exp.1	26	.04	5.91	< 0.001	100
Kwak et al. 2020, Exp.1b	44	.10	4.20	< 0.001	25
Lacey et al. 2020, Exp.1	46	.02	21.15	< 0.001	537
Peiffer-Smadja & Cohen, 2019, Exp.1	84	.08	10.70	< 0.001	24
Noise Band Exp., synthetic stimuli	69	.09	8.16	< 0.001	32

For each fit with the optimal Balance-Continuity model (or just Balance in the case of some studies^16,24^ Knoeferle et al., Exp. 1 and Kwak et al., Exp. 1b, respectively) displayed in Fig. 2, the corresponding *t* and *p* values for the estimation of explained variance *r*^*2*^ are provided.

Importantly, we tested whether the Balance x Continuity model could also efficiently predict, for non-speech stimuli, similar sound-shape associations as in the bouba-kiki effect with speech stimuli. More precisely, the goal was to assess whether artificial manipulations of spectral balance and temporal continuity parameters in non-speech stimuli could drive the participants’ associations of these non-speech stimuli with round or spiky shapes.

**Figure 3:**
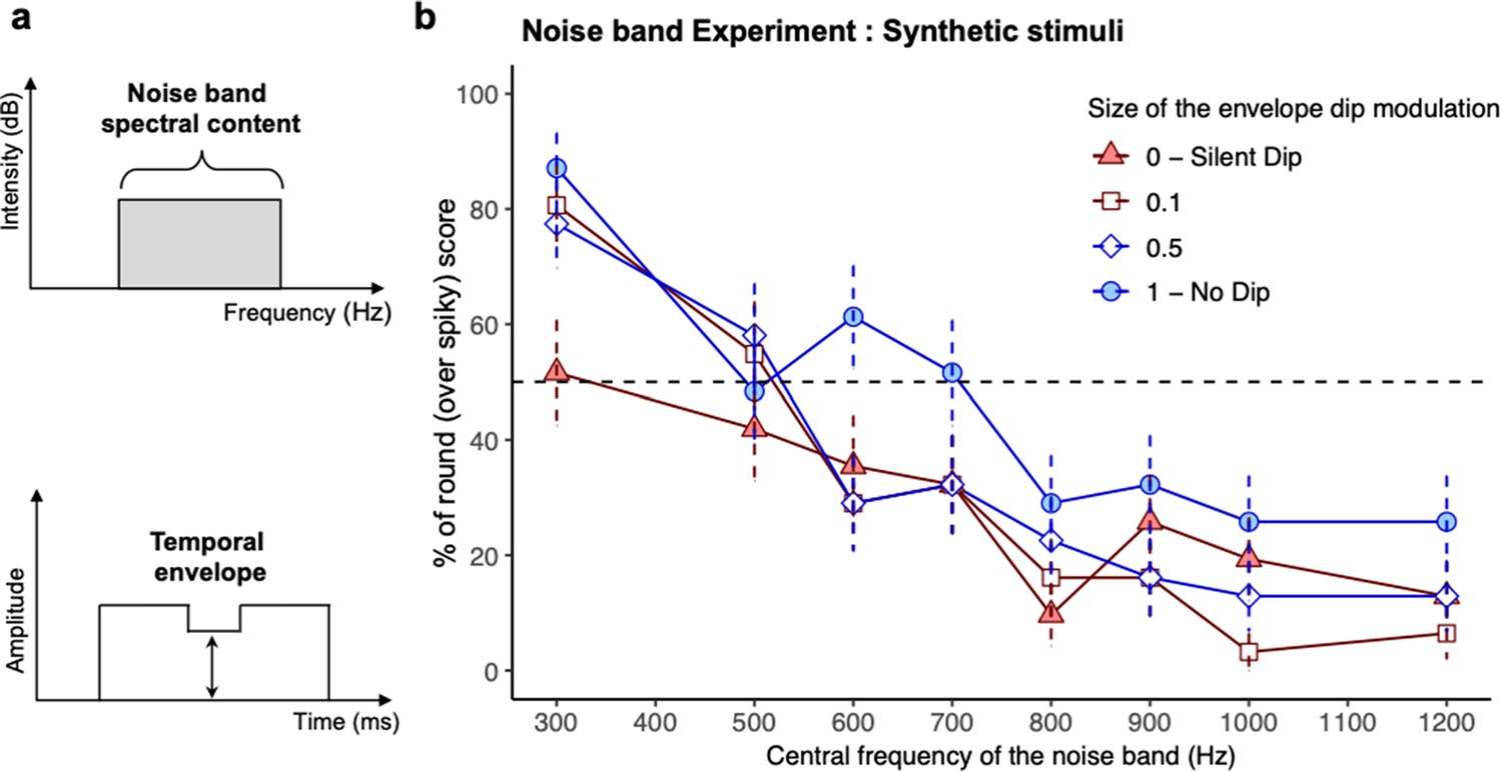
Stimuli manipulation and results from the Noise Band Experiment. (**a**) *Top*. Spectral content of the non-speech stimuli in the Noise Band Experiment made of bands of white noise, centered at values varying from 300 Hz to 1,200 Hz (Central frequency). *Bottom*. Amplitude modification of the stimuli to manipulate temporal continuity. Non-speech stimuli were 500 ms long and modulated by an envelope containing a dip from 225 to 275 ms, at an amplitude taking 4 possible values at 0, 0.1, 0.5 and 1 relative to the maximum value (Dip amplitude: 0 meaning a silent dip, and 1 meaning no dip at all). The amplitude was constant from 0 to 225 ms and from 275 to 500 ms. (**b**) Experimental results for the non-speech stimuli. The percentage of round (over spiky) score is averaged across participants as a function of the central frequency of the noise band and the size of the envelope modulation dip. Error bars are standard error from the mean.

According to the simulations from the Balance x Continuity model obtained for speech stimuli from pre-existing data, round scores should increase as non-speech stimuli display lower-frequency spectra and more continuous envelopes. We developed a new corpus of synthetic non-speech acoustic stimuli. We used white-noise bands modulated in amplitude, deliberately manipulating two parameters. We manipulated their spectral balance (called “Experimental_Balance” in the following) by controlling the central frequency of the band (Fig. 3a, top). We also manipulated their temporal continuity (called “Experimental_Continuity” in the following) by controlling the amplitude of a temporal dip inside the stimulus (Fig. 3a, bottom). Then, in a novel experiment (Noise Band Experiment, for more details, see "Methods"), we tested the tendency of adult participants to associate these stimuli with either round or spiky shapes using the same forced choice task as in previous bouba-kiki studies^6,9^.

Each participant’s response was coded as 1 for a choice for round shape or 0 for a choice for spiky shape. We then used a logistic mixed-effects model to analyze these non-averaged data with R^27^ and the “lme4” package^28^ (alpha level: 0.05, two-tailed). Experimental_Balance (300, 500, 600, 700, 800, 900, 1000, 1200 Hz) and Experimental_Continuity (0, 0.1, 0.5, 1) were within-participant fixed factors. The Experimental_Balance in the value 300 and the Experimental_Continuity in the full discontinuity (0) condition were used as intercepts. As predicted, results presented in Fig. 3b revealed a significant influence of Experimental_Balance (Estimate = -0.07, SE = 0.009, *t* = − 7.33, *p* < 0.001), indicating that stimuli with low-frequency spectra were more often mapped onto round shapes whereas stimuli containing high-frequency spectra were more often mapped onto spiky shapes. Still according to our predictions, results also revealed a significant influence of Experimental_Continuity (Estimate = 27.3, SE = 13.7, *t* = 2, *p* = 0.046), showing that continuous acoustic stimuli were more often associated with round shapes than discontinuous stimuli. Interaction between these factors was not significant (all *t* < 1). Simulations showed that the experimental data from the Noise Band Experiment were also well captured by the Balance x Continuity model (mean *r*^*2*^ = 69%, see Table 1).

Taken together, these results demonstrate that the bouba-kiki effect applies to non-speech acoustic stimuli as well as to speech utterances^29^. More importantly, they clearly confirm the predictive power of spectral balance and temporal continuity in the sound-shape associations of the bouba-kiki effect, capturing most of its universal phenomenology.

### Solving the second problem: explaining the cognitive mechanisms of the bouba-kiki effect phenomenology

The goal of this section is to provide a mechanistic framework for the bouba-kiki effect. More precisely, we determine how acoustic spectral balance and temporal continuity can generate in perceivers systematic associations with visual roundness/spikiness.

#### Can one hear the shape of a drum?

The title of this section is the exact title of a famous paper published by the mathematician Mark Kac^30^. In this paper, Kac mathematically demonstrates that the spectrum of a resonant two-dimensional surface, a “drum”, depends on its size, but also, for a fixed size, on the shape of its contour (see Supplementary Information, for more details). To be precise, he shows that the distribution of the frequency resonance modes of the sound of a drum is driven by both the area (size) and the perimeter of the surface. Classically, the larger the area the lower the mode frequencies: large objects produce low frequency sounds. But also, for a fixed drum area, the smaller the perimeter, the lower the mode frequencies and thus, the lower the spectral balance of the drum. Crucially, for a given surface area, the shape minimizing the perimeter is a circle: thus, the rounder the shape of a drum, the smaller its perimeter and the lower its spectral balance. Conversely, increasing the spikiness of a drum increases its perimeter, making its spectrum expand towards higher frequencies. In our view, one solution to the bouba-kiki effect enigma lies in this mathematical (and thus physical) fact, largely overlooked by researchers working on sound symbolism: round objects have overall lower spectral balance than same-size spiky objects.

We posit that perceivers are sensitive to this audiovisual regularity, which would drive the bouba-kiki effect.

**Figure 4:**
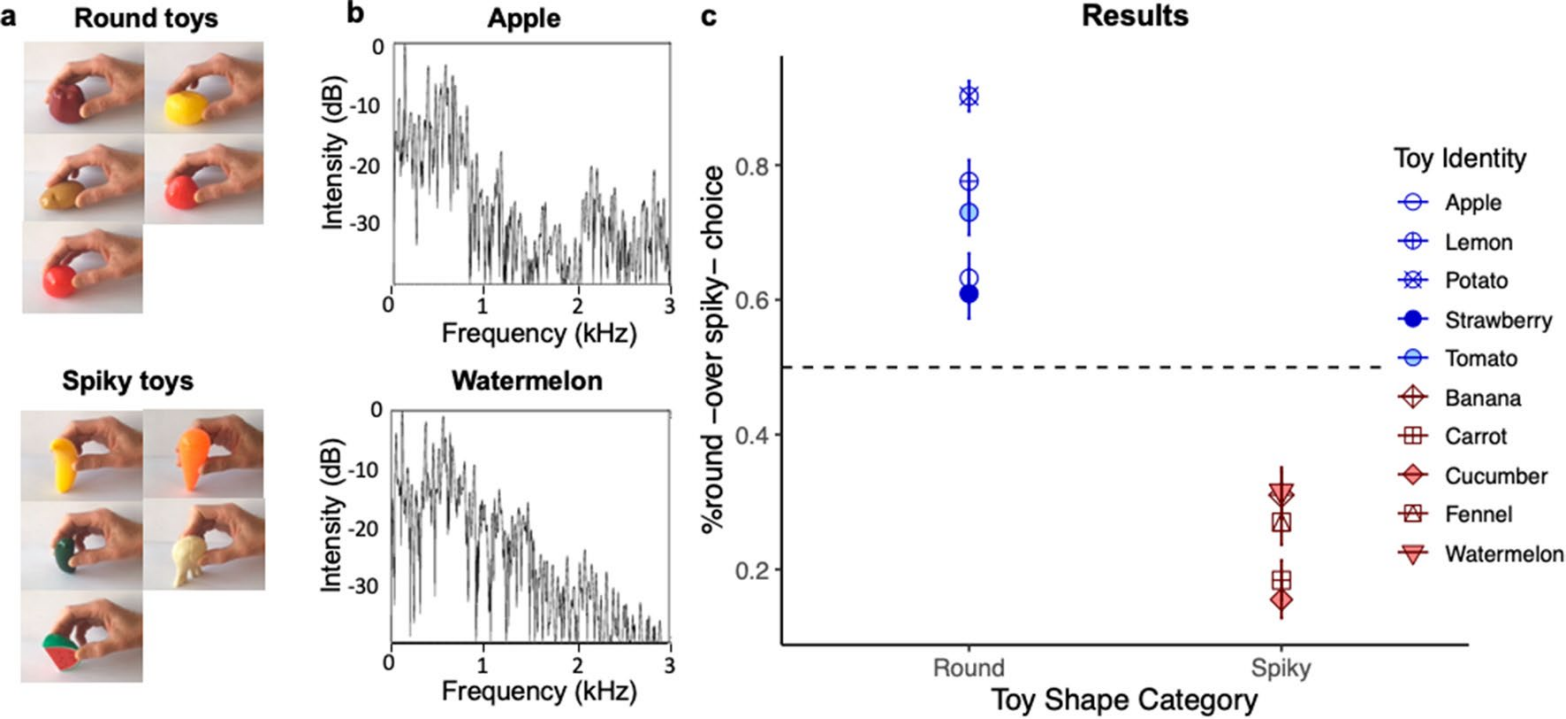
Stimuli and results from the Beating Toys Experiment. (**a**) Images of the visual stimuli (children’s plastic toy reproductions of fruits and vegetables). (**b**) Typical frequency spectrum resulting from hitting the base of a round (apple) or the tip of a spiky (watermelon slice) object on a hard surface. The spectral balance is lower for the round as compared to the spiky object. (**c**). Percentage of round (over spiky) score averaged across participants as a function of each stimulus object and Toy Shape Category (from left to right, Round: Apple, Lemon, Potato, Strawberry, Tomato; Spiky: Banana (tip), Carrot (tip), Cucumber (tip), Fennel (tip), Watermelon slice (tip)). Error bars are standard error from the mean.

To test this hypothesis, we designed an online forced-choice auditory experiment in which participants were presented with sounds produced by either a round or a spiky object and had to decide whether the object was actually round or spiky (Beating Toys Experiment, see "Methods", for more details). To do so, we selected ten similar-size ordinary objects, five rather round and five rather spiky, from a set of children’s toys (plastic fruits and vegetables, Fig. 4a). We recorded the sound produced by each of them when mildly hitting their base (for round objects) or their tip (for spiky objects) on a plane hard surface. We observed that they indeed differed by the spectral balance of their resonance spectrum, round objects producing lower-frequency sounds (see Fig. 4b, for examples of typical resulting sound spectra). The resulting sounds were all normalized in terms of intensity and similar in terms of duration (200–220 ms). At each trial, each object sound was presented together with two images (one of a round and one of a spiky toy) presented side by side. Participants had to match each sound to one of the two toy images.

Results are presented in Fig. 4c. Data coding and pre-processing were the same as in the Noise Band Experiment. We then applied a linear model analysis on the round scores averaged across stimuli using the “lm” function in R^27^ (alpha level: 0.05, two-tailed). Object Shape Category (Round, Spiky) was a within-participant fixed factor. Results revealed a significant influence of Object Shape Category on round scores (Estimate = -48.3, SE = 4.8, *t* = − 10.02, *p* < 0.001), indicating that participants could successfully identify the shape of the object by hearing its sound. To better assess the strength of the effect, we estimated the percentage of variance *r*^2^ explained by the factor Object Shape Category, by computing the squared correlation between the response variable and the predicted values, revealing a large effect size (*r*^2^ = 64%). In other words, participants were able to exploit the correspondence between sounds and shapes, with their judgments in agreement with Kac’s round-spiky property.

#### Can one hear the shape of a rolling drum?

The second parameter we identified influencing the bouba-kiki effect is temporal continuity. We posit that the explanation of this second factor also lies in another very simple physical fact. Round objects, when rolling, have a more continuous movement trajectory than spiky objects, producing sounds with smooth fluctuations of their acoustic envelopes. Alternatively, spiky objects yield more discontinuous trajectories than round objects, resulting in faster and larger fluctuations of their acoustic envelopes. This cross-modal regularity between shape and temporal continuity is also an inherent property of the physical world. We then tested whether perceivers are sensitive to this other cross-modal regularity.

**Figure 5:**
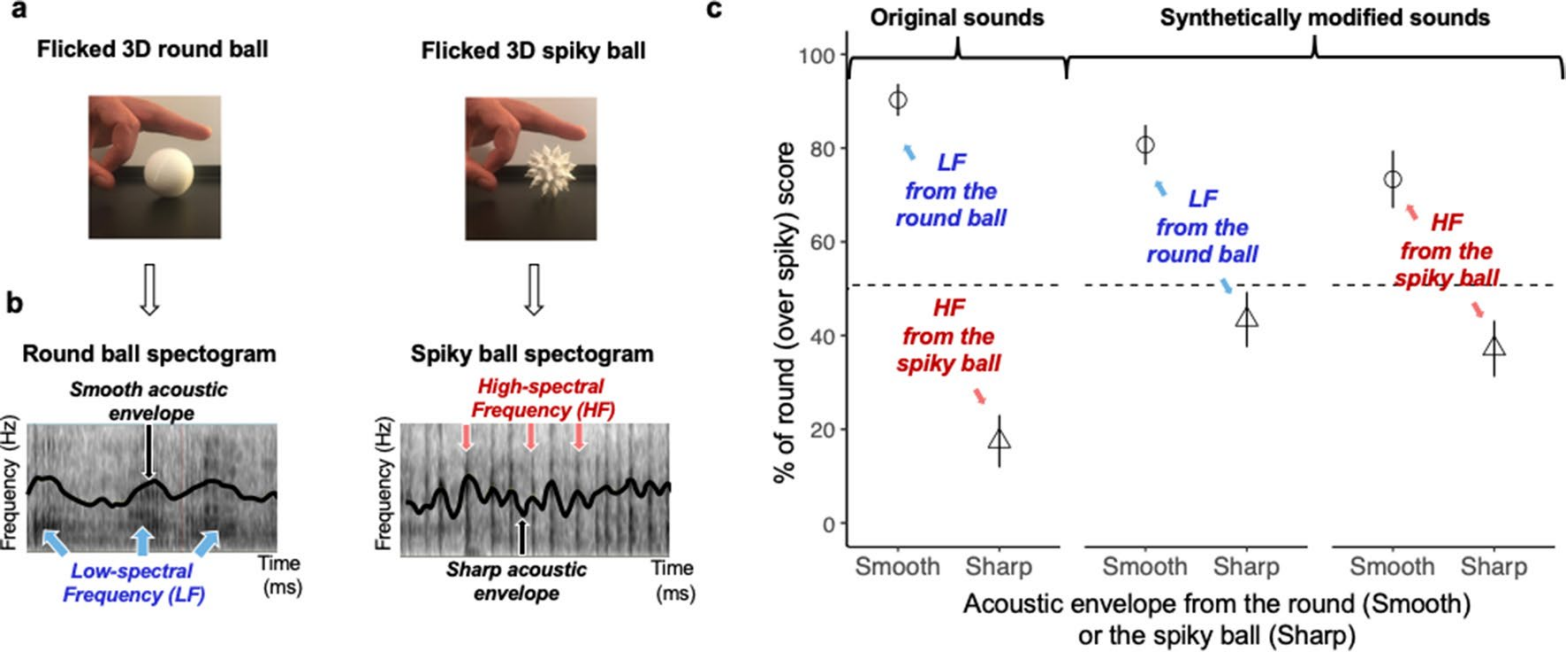
Stimuli and results from the Rolling Balls Experiment. (**a**). Picture of the two 3D balls (one round, one spiky) propelled by a flick on a plane shelf used in the forced-choice task. The balls were made with a 3D printer. (**b**) Examples of spectrograms of two original sounds produced by the round and the spiky balls rolling on a shelf. The acoustic envelope, drawn in black, is smoother for the round ball. Darker areas represent more intense low- (for the round ball) or high- (for the spiky ball) frequency bands. (**c**) Experimental round score averaged across participants for the Rolling Balls Experiment. These scores are represented as a function of Acoustic Envelope (Smooth—circles vs. Sharp—triangles) and Spectral Balance (Low-Frequency, LF, in blue vs. high-frequency, HF, in red) for the original and the synthetically modified sound stimuli. Big circles and triangles show overall means. Error bars are standard errors.

To test this hypothesis, we conducted one last online forced-choice experiment, asking participants to recover the shape of a rolling object, by listening to its acoustic envelope (Rolling Balls Experiment). We used a 3D-printer to construct two similar-volume 3D balls: one perfectly round sphere and one sphere with spikes (see Fig. 5a). We recorded several instances of the sounds (original sounds) produced when flicking each ball to roll on a hard plane surface, made of plastic or wood. Unsurprisingly, recordings showed a smoother acoustic envelope for the rounder ball (e.g. Figure 5b). We then presented these original sounds and synthetically modified sounds. To create the synthetically modified sounds, we applied the original temporal envelopes either from the round or the spiky balls to spectrally controlled sounds with either a low-frequency spectrum from the round ball or a high-frequency spectrum from the spiky ball (see "Methods" for more details). At each trial, each sound was presented together with two images, one of the round and one of the spiky ball. Participants had to match each sound to one of the two ball images.

Results are displayed in Fig. 5c. Data coding and pre-processing were the same as in the Noise Band Experiment. We then used a mixed-effects model to analyze these non-averaged data with R^27^ and the “lme4” package^28^ (alpha level: 0.05, two-tailed). The data for the original sounds and the synthetically modified sounds were analyzed separately. For the original sounds, Ball Shape (Round, Spiky) was the single within-participant fixed factor. For the synthetically modified sounds, Acoustic Envelope (Smooth from the round ball, Sharp from the spiky ball) and Spectral Balance (Low-Frequency from the round ball, High-Frequency from the spiky ball) were within-participant fixed factors. Initially, rolling Surface Material (wood, plastic) was also declared as a random factor, but it did not significantly increase the variance accounted for and was thus excluded from the final models. The Smooth Acoustic Envelope and Low-Frequency conditions were used as intercepts. For the original sounds, results revealed a significant influence of Ball Shape (Estimate = − 71.7, SE = 3.3, *t* = − 22.1, *p* < 0.001), indicating that participants could successfully identify the shape of the ball by hearing it rolling on a hard surface (the percentage of explained variance *r*^2^, computed as for the Beating Toys Experiment, displays a large effect size, *r*^2^ = 69%). For the synthetically modified sounds, results revealed a significant influence of Acoustic Envelope (Estimate = − 36.7, SE = 4.3, *t* = − 8.7, *p* < 0.001), indicating that participants could successfully identify the shape of the ball by only hearing its envelope (effect size moderate but clearly non-negligible, *r*^2^ = 31%). The effect of Spectral Balance was tendential (Estimate = − 7.2, SE = 4.3, *t* = − 1.7, *p* = 0.08) suggesting that in this specific situation, its role was probably concealed by the massive effect of Acoustic Envelope. In other words, results clearly show that participants were able to exploit the dynamics (temporal continuity) of the acoustic envelope to determine the shape of the rolling ball it was coming from, for both original sounds and their spectrally-controlled synthetical counterparts.

In sum, we posit that the mechanisms of the bouba-kiki effect result from two simple physical properties of round vs. spiky objects in the environment. We showed that these physical properties influence the spectral balance and the temporal continuity of the sound produced by these objects. Last, we demonstrated that human adults can use this intuitive audiovisual physical knowledge to identify the shape of novel objects, by hearing them hitting or rolling on a hard surface.

## Discussion

In this paper, we provide a coherent explanation of the bouba-kiki effect enigma, resolving the two problems raised in the introduction. We first identified two universal acoustic parameters involved in this effect (spectral balance and temporal continuity). We then unveiled the underlying cognitive mechanisms at play in this phenomenon. A sound is perceived round or spiky because it is likely to be produced by a round or a spiky object hitting or rolling on a hard surface. Round objects, as compared to similar-size spiky objects, have lower-frequency acoustic resonance modes resulting in sounds with more energy in the low-frequency part of the acoustic spectrum, explaining the role of spectral balance. When rolling on a hard surface, they also produce smoother trajectories than their spiky counterparts, thus producing sounds with more continuous acoustic envelopes, explaining the role of temporal continuity. Human participants appear to be sensitive to these physical properties (see Supplementary Information for more discussion).

The two-components phenomenological model of the bouba-kiki effect appears very efficient, explaining a large part of the variance of experimental data. This is even more impressive considering that it is only poorly fitted to the precise acoustic content of the stimuli and specific experimental paradigm of each experiment. Indeed, the speech stimuli were considerably diverse in terms of language used, phonological structure and content, speaking style and gender, word length, etc. (see Table 2 and further discussion in the Supplementary Information). Yet, we acknowledge that additional acoustic factors likely intervene in the fine tuning of the bouba-kiki effect, particularly concerning pitch^13^, or the vocal source in relation to its shimmer^10^. Orthographic factors together with phonotactic and phonological constraints also play a role in this effect and in sound symbolism in general (see^12,23,31^ for recent overviews). More globally, the bouba-kiki effect may be part of a larger system of sound-to-semantic associations which could involve higher-order factors such as arousal (termed “activity” in^32^).

**Table 2:** Presentation of the data and speech stimuli of the 10 published experiments assessed in the computational phenomenological model.

Study (date)	Exp. num.	Participants (N)	Speech stimuli description—examples		Visual shapes (N)	Proc.	Dependent variable (DV)	Round score	Stim. & data availability
Ahlner & Zlatev (2010)	1	Swedish-speaking (20)	Natural speech, 16 CVCV, 2 V, 8 C, ADS, male speaker	/lili/, /mumu/, /titi/, /tutu/	Round (1), Spiky (1)	FCT, onsite	Percentage of round score [0%; 100%]	DV	Not shared publically
Bottini, et al. (2019)	2	Native Italian (18)	Natural speech, 240 CVCVCV, 4 V, 15 C, ADS, male speaker	/mulomu/, /tekiti/, /melime/, /tukotu/	No shape	FCT, online	Percentage of round score [0%; 100%]	DV	https://osf.io/yc4ud/
D’Onofrio (2014)	1	Native English (170)	Natural speech, 24 CVCV, 4 V, 6 C, ADS, male speaker	/pipe/, /pepi/, /bubɑ/, /bɑbu/	Round (2), Spiky (2)	FCT, online	Percentage of round score [0%; 100%]	DV	Not shared publically
Fort, et al. (2015)	1	Native French (24)	Natural speech, 32 CVCV, 9 V, 4 C, IDS, female voice	/lumu/, /mili/, /koto/, /teke/	Round (16), Spiky (16)	FCT, onsite	Percentage of round score minus 50% [− 50%; 50%]	DV	https://osf.io/v9xyd/?view_only=2d207fc228684f1d8ac96ac97f57759c
	2	Native French (24)	Natural speech, 32 VCV, 9 V, 4 C, ADS, female speaker	/ulu/, /imi/, /yky/, /ata/					
	3	Native French (23)	Natural speech, 56 CVCV, 4 V, 15 C, IDS, female voice	/pupo/, /popu/, /kike/, /keki/	Round (14), Spiky (14)				
Knoeferle, et al. (2017)	2	Native English (30)	Natural speech, 100 CV, 5 V, 20 C, ADS, female speaker	/wa/, /ju/, /lo/, /re/	Round (5), Spiky (5)	LRT, onsite	Mean rating score [1-round; 5-spiky]	5 minus—DV	https://osf.io/xsejv/
Kwak, et al. (2020)	1b	Native Korean (40)	Synthesized speech, High-front, Low-front, High-back and Low-back 25 V , ADS	Cf. paper	Round (1), Spiky (1)	FCT, onsite	Percentage of spiky score [0%; 100%]	100 minus—DV	Not shared publically
Peiffer-Smadja & Cohen (2019)	1	Native French (14)	Natural speech, 24 CVCV, 4 V, 6 C, ADS, male speaker	/keti/, /mujo/	Round (4), Spiky (4)	FCT, onsite	Percentage of round score [0%; 100%]	DV	Not shared publically
Lacey, et al. (2020)	2	Native English (31)	Natural speech, 537 CVCV pseudowords, 7 V, 15 C, ADS, female speaker	/kotu/, /bubo/, /kite/, /bibe/	No shape	LRT, onsite	Mean Rating scores [1-rounded); 7-pointed)]	7 minus—DV	https://osf.io/y9zjc/

The experiment number matches the one from the original publication. The Experimental “Round Score” in the second last column is the one used in all simulations and analyses of the present paper. *FCT* forced choice task, *LRT* likert rating task, No Shape Participants judged how each speech stimulus sounded, *DV* dependent variable, Round Score assessment (averaged across participants) used in each study, *ADS* adult-directed-speech, *IDS* infant-directed-speech, *Proc*. procedure.

Importantly, the mathematical analysis by Kac^30^ shows that first size and then, when the size parameter is neutralized, contour shape of a drum influence the spectral balance of its sound. The link between these two mathematical properties can be interpreted in simple intuitive terms. A spiky shape has locally small parts (spikes), providing local spots with higher spectral components, thus shifting the overall spectral balance. This is what happens when hitting a plastic watermelon slice on its tip (Beating Toys Experiment). Note that this kind of object can sound either round or spiky depending on the part of the object that is hit on a hard surface. The spiky character feature here must be appreciated globally, as an average of all local aspects of the object. Conversely, a round shape is locally always as large as possible, resulting in a lower spectral balance. This could result in a possible confound between these two factors. However, experimental studies suggest otherwise. For instance^16^ showed in their Experiment 1 that size estimation is rather related to differences in vowel height (in the first formant region) and vowel duration. Other findings suggest that pitch and intensity also play a role in size estimation^33,34^ with a number of consequences in terms of vocal exaggeration of body size and deceptive communication signals (for an overview see^35^). When hearing a sound, it is thus possible that first, a gross estimation of size operates, mainly provided by vowel and prosodic features. Then, more subtle processes for shape estimation can occur, based on spectral balance and temporal continuity cues. Another possibility is that the use of same-size visual shapes and explicit shape judgement tasks in bouba-kiki paradigms drive participants to fully interpret spectral balance only as a cue for estimating shape**.**

Since the sensitivity to audiovisual regularities relating shape with spectral balance and with temporal continuity is likely rooted in physical properties of objects, these regularities could be learned during development. In the context of the debate contrasting innate vs acquired mechanisms for the emergence of sound symbolism^36–42^, we provide a novel and decisive argument. Indeed, we demonstrated that the bouba-kiki effect is not speech specific and relies on universal statistical regularities of object sounds that are present (and potentially frequent) in infants’ perceptual input. Similarly, audiovisual correspondences involving pitch and visuospatial height have been shown to be rooted in regularities of the physical environment^43^.

One could then hypothesize that language evolution itself could have been influenced by the regularities of environmental scenes. In line with this hypothesis^14^ found in the adult English lexicon that round or curvy objects are slightly more often associated with labels containing voiced consonants with low frequencies (e.g. /b/ in bouba) than with unvoiced high frequency ones (e.g. /k/ in kiki). Other studies also showed that the audiovisual correspondence underlying the bouba-kiki effect favors word learning and language generation^4,14,42,44^. Sensitivity to these audio-visual regularities could be learned through ontological development as a cross-modal part of the intuitive physics system and then play a role in or even bootstrap language learning.

Last but not least, if the bouba-kiki effect is actually learned from non-speech audiovisual regularities available in the physical environment, it could also be learned by animals. So far, two recent studies on adult apes suggests that they are not sensitive to the classical bouba-kiki effect^45,46^, while other studies with adult chimpanzees suggest that they are sensitive to other audiovisual correspondences^47,48^. Yet, the apparent lack of sensitivity to the bouba-kiki effect could be due to several reasons. First, the use of abstract 2D pictures of the shapes in both studies have been shown to be difficult to interpret in a 3D mode by animals^49^. In our interpretation of the mechanisms behind the bouba-kiki effect this parameter is crucial as only real 3D objects can produce sounds when hitting or rolling on the ground. Second, the use in these studies of a great number of novel speech items (20 pseudowords) and of human speech stimuli that are infrequent for apes – even though they have been trained to some of them in one of these studies^46^ – could have prevented them to spontaneously form an audiovisual association with a corresponding visual shape. Indeed, spontaneous cross-modal matching tasks have been proven to be difficult for apes, see^48^ for an overview. Our findings that the bouba-kiki effect can be produced by simple non-speech sounds provides novel perspectives for exploring the origin of this effect. As hypothesized for human ontological development, non-human primates could be sensitive to simpler audiovisual associations between non-speech sounds (e.g. high- vs. low- spectral frequency noise band) and 3D object shapes, after experiencing and learning the same audiovisual regularities in their environment.

## Methods

### Solving the first problem: a universal phenomenological model of the bouba-kiki effect

#### Data gathering for the meta-analysis

Simulations were applied to the experimental data contained in eight publications, providing altogether 10 experiments (Table 2). We followed the PRISMA statement^50^ for selecting and reporting the studies to be included in our meta-analysis. We decided to include articles if they were testing typically-hearing and typically-sighted adults, assessing whether participants would associate certain auditory pseudo-words rather to round or to spiky shapes, thus, testing both round (or bouba-type) and spiky (or kiki-type) cross-modal correspondences.

The selected studies involved explicit rating of the roundness or the spikiness of pseudowords, using a binary choice or a quantitative scale (7-points Likert rating tasks) between a round and a spiky shape. Note that we do not directly address here the possibility that round and spiky scores could vary independently. Other work^51^ showed that separate quantitative Likert rating tasks of roundedness and spikiness mirror both one another: words rated higher on the rounded scale were rated lower on the spiky scale and vice versa. Moreover, the same study also showed that the responses given in the two-alternative forced choice task between a round and a spiky shape are highly correlated with the ones given for the same stimuli in 7-points Likert rating tasks.

To explore the acoustic components at play in the bouba-kiki effect, we selected studies testing a large number of speech stimuli (N > 10) presented auditorily (excluding written presentations) and testing each speech stimulus only once (e.g. excluding implicit association and word learning tasks). We then requested from the first and/or last authors of 14 original articles their acoustic stimuli and the mean score (round vs. spiky choices averaged over all the participants of a given experiment) associated with each speech stimulus. Authors of eight articles kindly provided their stimuli and data in a usable format for the present paper, for a total of 10 experiments. Each consisted in a set of auditory speech stimuli with their corresponding round score rating averaged across participants (defined in Table 2, “Dependent Variable” and “Experimental round score” columns).

#### Balance x Continuity model description

Each acoustic speech stimulus of each experiment was processed in the computational phenomenological model displayed in Fig. 1. The model was defined as follows. The stimulus was fed into a gammatone filter-bank made of 64 filters from 50 to 20,000 Hz^52^. The resulting time–frequency representation was then processed in two separate pathways, respectively computing Balance and Continuity. In the Balance pathway, the mean spectrum *S*_*1..64*_ was computed by averaging spectral frames over time for each of the 64 frequency channels. The spectral balance value was then obtained by subtracting the summed energy values in frequency channels above and below a fixed boundary *b*:

1 $$Balance=\sum_{i=1}^{b}{S}_{i}-\sum_{i=b+1}^{64}{S}_{i}.$$

The value of the boundary *b* was varied between channels 20 (800 Hz) and 30 (1800 Hz) corresponding to the middle range of the acoustic spectrum around 1 kHz. This enabled to compare low-frequency regions (i.e. below 800–1800 Hz) typical of phonemes associated with round shapes (e.g. the voicing bar or nasal murmur for voiced or nasal stops, the burst spectrum for bilabial or labiodental consonants or the region of the first two formants for back vowels), with high-frequency regions above 800–1800 Hz, typical of phonemes associated with spiky shapes (e.g. the burst spectrum for coronal consonants or the high second or third formants for front vowels).Temporal analysis began by computing the summed energy *E*_*1..T*_ across all 64 frequency channels for each temporal frame from 1 to T (T being the stimulus duration in number of frames). From there on, the temporal continuity index was obtained by comparing the minimum to the maximum value inside the stimulus:

2 $$Continuity=\mathrm{log}\left(\frac{{min}_{t=1..T}{E}_{t}}{{max}_{t=1..T}{E}_{t}}\right).$$

Importantly, this index was computed only for di- or tri-syllabic speech stimuli, containing a consonant phoneme in between a sequence of two vowel phonemes. This computation enabled the capture of the magnitude of envelope decrease during the consonant between two peaks of intensity associated with the two vowels. This was possible for all experiments except for the Consonant–Vowel stimuli used in the study^16^, Exp. 1 and the Vowel-only stimuli used in the study^24^, Exp. 1b (see Table 2 for more details). Finally, the round scores for all stimuli of a given experiment were linearly predicted from the values of Balance and – if available – Continuity. We conducted separate linear regressions for each experiment between the model’s round score predictions and the experimental round score as a function of the value of the boundary *b* between channels 20 and 30. The *b* value maximizing explained variance was selected for providing predictions of the Balance x Continuity model in Table 1 and Fig. 2. The pattern of variations of *r*^*2*^ values with the boundary *b* across all experimental data sets is further discussed in the Supplementary Information. These analyses were conducted using R^27^ and the work package “lm”.

#### Experiments

All the experiments in this manuscript including human participants followed the General Data Protection Regulation (GDPR), and was approved by a local ethical committee (CERGA, “Comité d'Ethique pour la Recherche, Grenoble Alpes”*, Ethical Research Committee Grenoble Alpes* n° 2020-07-01-1). This means that methods were performed in accordance with the relevant guidelines and regulations for testing human participants in the European Union. Notably, informed consent was required from all participants before participating in the study.

#### Noise Band Experiment

##### Participants

The 31 participants were all anonymous French native speakers recruited and paid for their participation through the online platform Prolific (mean age: 26.6 years, range: 18–49). Five additional participants were rejected from the final sample for failing at one or more catch trials (3) or for completing the study in less than 5 min (2). None of them reported a known history of hearing, vision or language impairment.

##### Acoustic non-speech stimuli

Thirty-two noise stimuli were created using bands of white noise, 500-ms long and centered at eight possible frequency values (Experimental_Balance) between 300 and 1200 Hz within the set [300, 500, 600, 700, 800, 900, 1000, 1200], with a sampling frequency at 44,100 Hz (Fig. 3a, Top). These noise bands were created in Praat^53^ by filtering white noise by a formant filter centered at the target frequency between 300 and 1200 Hz and with a bandwidth equal to a tenth of the center frequency. To manipulate temporal continuity, their envelope amplitude was modulated from 225 to 275 ms by a dip taking 4 possible values (Experimental_Continuity) at 0, 0.1, 0.5 and 1 relative to the maximum value (0 meaning a completely discontinuous silent dip, and 1 meaning no dip at all, complete continuity, see Fig. 3a, Bottom). The remaining amplitude envelope was fixed at 1 from 0 to 225 ms and from 275 to 500 ms.

##### Visual shapes

The visual shapes were the same as the ones used in^9^, Exp. 2.

##### Procedure

The task was performed online on the platform Qualtrics. Participants were required to sit in front of a computer or a phone monitor in a quiet location and were strongly encouraged to listen to the acoustic stimuli through headphones. They were able to test their sound quality on two acoustic examples before the test. For each trial, one round and one spiky shape appeared on the screen, against a white background. Participants could listen to the acoustic stimuli by clicking on a dedicated play button. At each trial, participants had to choose which of the two shapes they felt that the acoustic stimulus referred to more closely. The whole experiment contained 32 test trials and two additional catch trials in which the acoustic stimuli was a recording explicitly asking participants to select either the round or the spiky shape. Those catch trials were there to ensure that participants were paying attention to the acoustic stimuli. Each acoustic stimulus was matched with a pair of round-spiky shapes. Round and spiky shapes appeared an equal number of times on the left and right sides of the screen, and the order of trials was randomized.

#### Beating Toys Experiment

##### Participants

Twenty-nine anonymous French native adult participants were recruited and paid for their participation through the online platform Prolific (mean age: 28 years, range: 19–42 years). No additional participant was rejected, that is all participants from the final sample succeed at catch trials and completed the study in more than 5 min. None of them participated in the Noise Band Experiment nor reported a known history of hearing, vision, or language impairment.

##### Acoustic and visual stimuli

We recorded a total of 30 sounds produced by five round and five spiky objects (3 sound occurrences per object), when gently hitting them on their base (round objects) or on their tip (spiky objects) on a hard and plane surface. Sounds were all normalized in terms of intensity and similar in terms of duration (200–220 ms). The high-frequency part of the spectra for sounds produced by spiky objects was clearly more intense than for sounds produced by round objects. Conversely, the low-frequency part of the spectra of sounds produced by round objects was clearly more intense than by spiky objects. Typically, there was more energy below 500 Hz for all round objects compared with spiky ones, and more energy between 500 and 2000 Hz for all spiky objects compared with round ones. Examples of spectra corresponding to the sound of a round or a spiky object are displayed in Fig. 4b. The objects were plastic fruits and vegetables from a set of children toys. Pictures of these objects being hit on a hard surface either on their tip (spiky objects) or on their base (round objects) were used as representations of these objects in the forced choice task (Fig. 4a).

##### Procedure

The task was performed online on the platform Qualtrics, following the General Data Protection Regulation (GDPR), and was approved by a local ethical committee (CERGA n° 2020-07-01-1). Participants performed the task online in the same conditions and following the same general forced choice procedure as in the Noise Band Experiment. At each trial, participants had to choose between the image of a round object hit on its base or a spiky object hit on its tip, that they felt was more likely to produce the single sound they were presented with. The whole experiment contained 60 test trials and four additional catch trials. Each acoustic stimulus was repeated twice and matched with its corresponding object (presented once on the left side of the screen, once on the right) and with two different distractors (one distractor per trial). Round and spiky objects appeared an equal number of times, and an equal number of times on the left and right sides of the screen. The order of trials was randomized among participants.

#### Rolling Balls Experiment

##### Participants

Thirty anonymous French native adult participants were recruited and paid for their participation through the online platform Prolific (mean age: 21.9 years, range: 18–32). No additional participants were rejected from the final sample, that is all participants from the final sample succeed at catch trials and completed the study in more than 5 min. None of them reported a known history of hearing, vision, or language impairment nor participated in the previous experiments.

##### Acoustic and visual stimuli

Using a 3D printer, we first printed two same-size 3D balls: one round and one spiky. We then recorded the sound produced by these two balls rolling on a hard wood or plastic surface (see Fig. 5a). This provided 12 original sounds, from two objects (round and spiky balls) x two surface materials (hard wood and hard plastic) and 3 instances per object and surface material per condition (see examples of corresponding envelope fluctuations in Fig. 5b). To control for differences in frequency spectrum between round vs. spiky objects, we estimated the mean spectrum of all sounds respectively produced by round objects and spiky objects. We used the Praat Speech_Shaped_Noise script^53^ to generate two noise stimuli “shaped” by these mean values for round and spiky frequency spectra. We then estimated the acoustic envelope of each of the 12 original stimuli using^54^ and we modulated in time the two shaped-noise-spectra stimuli by these 12 envelopes. We obtained a total of 24 additional synthetically modified stimuli with two spectral shapes (one low-frequency from the round ball and one high-frequency from the spiky ball), with the temporal envelope from the sounds produced by the round or the spiky ball rolling. This gave rise to a final set of 36 acoustic stimuli (12 original ones plus 24 additional synthetic ones) along with two visual stimuli (picture of the two balls). Sounds were similar in duration (around 200 ms), and they were all normalized in intensity. Unsurprisingly, a smoother acoustic envelope was found for the round ball. Indeed, local intensity variations are both large and rapid for all spiky trajectories (greater than 8 dB within less than 40 ms) and smaller and slower for all round trajectories (less than 6 dB within more than 40 ms), e.g. Fig. 5b.

##### Procedure

The task was performed online on the platform Qualtrics, following the General Data Protection Regulation (GDPR), and was approved by a local ethical committee (CERGA n° 2020-07-01-1). Participants performed a forced choice online in the same conditions and following the same general forced choice procedure as in previous experiments. At each trial, participants had to select which object, between the round and the spiky ball, was more likely to produce a sound they were given to hear. Each sound was presented in a random order, associated to the pair made of the spiky and the round images from Fig. 5a. The task of the participants was to select the object (round or spiky) corresponding to the sound. The precise instruction was the following: “At each trial, you will listen to a sound obtained by rolling one of these two balls on a hard plastic or wooden shelf, propelled by a flick. Your task is to tell if the sound corresponds more to the sound obtained by rolling the round or the spiky ball”. The whole experiment contained 36 test trials and two additional catch trials. Each acoustic stimulus was presented only once to each participant. Round and spiky balls appeared an equal number of times on the left and right sides of the screen. The order of trials was randomized among participants.

## Supplementary Information

Supplementary Information.

## Data Availability

Not all the authors of the experiments included in the meta-analysis shared their data publicly (cf. Table 2). Summary of available data, stimuli and script of the model, as well as the data, stimuli and scripts used in the Noise Band, Beating Toys and Rolling Balls Experiments are available in this osf: https://osf.io/v9xyd/?view_only=2d207fc228684f1d8ac96ac97f57759c.
