# Supplementary material for: Resolving the bouba-kiki effect enigma by rooting iconic sound symbolism in physical properties of round and spiky objects
Source: Sci Rep. 2022 Nov 10;12:19172. doi: 10.1038/s41598-022-23623-w (PMC9649795; doi:10.1038/s41598-022-23623-w)
Supplement: Supplementary file 1 — Supplementary Information. [file 41598_2022_23623_MOESM1_ESM.docx]

**Supplementary Information for**

Resolving the bouba-kiki effect enigma by rooting iconic sound symbolism in physical properties of round and spiky objects

Mathilde Fort^1&2^*, Jean-Luc Schwartz^3^

^1^Laboratoire de Psychologie et NeuroCognition, UMR 5105, Université Grenoble Alpes, Grenoble, France.

^2^ Centre de Recherche en NeuroSciences de Lyon, UMR 5292, Université Lyon 1, Lyon, France.

^3^ Grenoble Images Parole Signal Automatique, UMR 5216, Université Grenoble Alpes, CNRS, Grenoble INP, Grenoble, France.

*Corresponding author: Mathilde Fort

**Email:**  mathilde.fort@univ-grenoble-alpes.fr

**This file includes:**

Supplementary Information Text

Supplementary Information Figures S1 to S3

Supplementary Information References

**Other supplementary materials for this manuscript include the following:**

Link for datasets

Supplementary Information Text

**Solving the first problem: A universal phenomenological model of the bouba-kiki effect**

***A phonetic analysis of pre-existing experimental data on the bouba-kiki effect***

The published reports about the bouba-kiki effect in adults^1–13^ let emerge a consistent five-points (noted P_1..5_) pattern:

P_1_ Nasals as /m n/ and to a lesser extent voiced plosives as /b d g/ are rounder (i.e., more associated with round shapes) than unvoiced ones as /p t k/ (i.e., more associated with spiky shapes);

P_2_ Bilabials as /p b/ are rounder than coronals as /t d k g/;

P_3_ Liquids as /l/, laterals as /r/ and semi-vowels as /j w/ are mostly round;

P_4_ Fricatives as /f s v z/ are less spiky than unvoiced plosives;

P_5_ Back vowels such as /u o/ are rounder than front vowels such as /i y e/, whether they are lip-rounded or not.

A phonetic analysis provides a straightforward interpretation of this phenomenology. First, it appears that speech stimuli associated to rounder shapes have lower-frequency spectra. While all plosives are characterized by a closure period followed by an acoustic burst with relatively high frequency, the closure period is silent for unvoiced plosives but it is filled with a low-frequency component for nasals (with the “nasal murmur”) and for oral voiced plosives (with the “voicing bar”)^14^, compatible with P_1_. Labial plosives are characterized by “diffuse falling” spectra dominated by low frequencies, while coronals are characterized by “diffuse rising” or “compact” spectra dominated by mid or high frequencies^15^, explaining P_2_. Liquids, laterals and semi-vowels are characterized by strong energy in the low-frequency region of their spectrum^14^, explaining P_3_. As compared with higher-frequency front vowels, back vowels are characterized by a low second formant and a lower-frequency spectrum overall^14,16^, compatible with P_5_. Second, speech stimuli associated with rounder shapes display smoother fluctuations of their acoustic envelope. While both fricatives and unvoiced plosives have high-frequency spectra, fricatives are characterized by a smoother envelope^14^, elucidating P_4_. The low-frequency components during closure in nasals and oral voiced plosives mentioned above make their envelope smoother than unvoiced plosives^15^, reinforcing P_1_.

***Global model performances***

The Balance x Continuity model provides linear predictions of experimental data as a function of the value of the boundary *b* (see *Material and* Methods for a definition of *b)*. In the following we discuss this pattern of variation, assessed by the percentage of variance *r^2^* explained by the model for each *b* value. Fig. S1a displays this percentage as a function of the value of the boundary *b*. We observe that the explained variance *r^2^* varies with the value of the boundary *b* between as well as within experiments – particularly for ^2^, Exp. 1 and ^13^, Exp. 1b, where the Continuity predictor is not available. Crucially, *r^2^* reaches large values for each of the 10 experiments, ranging from 26 to 94% (see Table 1 and Fig. 2). Fig. S1b displays the *r^2^* explained by Balance as a function of the *r^2^* explained by Continuity, for the eight experiments for which both predictors were available. The *r^2^* values for each predictor were estimated by using a single predictor for predicting experimental data, with Balance set at its optimal value maximizing explained variance (Fig. S1a) for each corresponding experiment. Both predictors provide similar performance (for Balance*-*only, mean *r^2^=* 39%, range: 12-62%; for Continuity-only, mean *r^2^=* 44%, range: 10-93%). Interestingly, their *r^2^* vary considerably between experiments, with no correlation between the roles of the two predictors (Fig. S1b).

<Figure S1ab>

***Detailed model performances***

To better understand why the roles of Balance and Continuity vary so much within each experiment, we also analyze how the model behaves for each experiment, as a function of the nature of its phonetic content. To do so, this new round of analyses is based on grouping speech stimuli as a function of different phonetic classes. We then explore how averaged experimental round scores for each phonetic category depend on the corresponding Balance or Continuity values averaged for the same phonetic category.

In Exp.1-2 of one study^6^, the authors contrasted very round sonorant (S) consonants /l m/ with very spiky unvoiced stop (US) consonants /t k/ in various vocalic contexts. They evidenced a strong effect of consonants and a smaller – but significant – additional effect of vowels. Fig. 2 well displays that the very high performance of the Balance-Continuity model (94% of the variance in both cases) is actually driven by the existence of two groups of stimuli which correspond to the two consonantal classes S and US. Since these two consonantal groups are clearly separated in terms of both spectral balance and temporal continuity, for these two experiments, Continuity alone explains more than 90% of the experimental variance, and Balance alone explains more that 50% of the variance for any boundary value between channels 20 and 30 (Fig. S1).

Predictions for two studies^3,9^ are both extremely accurate, respectively with values *r*^2^ = 71% and *r*^2^ = 84% of explained variance (Fig. 2 and S1a), though with very different roles of Balance and Continuity (Fig. S1b). The study^3^ is focused on stop consonants, contrasting voiced vs. unvoiced plosives with various places of articulation and in front vs. back vocalic contexts. For such stimuli, Continuity only helps to contrast unvoiced with voiced stops. Balance however efficiently contrasts between voiced /b d g/ vs. unvoiced /p t k/ stops on the one hand, and bilabial /b p/ vs. coronal /d t/ or velar /g k/ stops on the other. Within each consonant class, Balance also contrasts front- (-fv) vs. back-vowel (-bv) contexts. This explains why in Fig. S2a1, Balance (for the optimal value of the boundary *b*) correlates closely with experimental round scores averaged across all stimuli associated with the 6 stop consonants /p t k b d b/, while Continuity in Fig. S2a2 only contrasts unvoiced vs. voiced stops with small correlation with experimental data. In contrast, Continuity plays a major role in one study^9^ opposing highly discontinuous unvoiced stops (/p t k/) with more continuous voiced fricatives (/ʒ/) and sonorants (l m/) (Fig. S2b2). Balance appears however as a poor predictor of experimental round score (Fig. S2b1).

<Figure S2>

Then comes a coherent group of four studies^1,4–6^: Exp. 2, Exp. 1, Exp. 1 and Exp. 3, respectively. These experiments all deal with similar material involving five consonant categories: Sonorants, Voiced or Unvoiced Fricatives, and Voiced or Unvoiced Stops. They are presented in two types of vowel contexts: front and back. The predictions for these four experiments are fairly accurate, respectively accounting for *r*^2^ = 59%, *r*^2^ = 48%, *r*^2^ = 46% and *r*^2^ = 38% of the experimental variance (Fig. 2 and S1a). These predictions are based on both Balance and Continuity (Fig. S1b). Fig. S3 shows that in all four cases, Balance (Fig S3, left column: from a1 to d1) is a more reliable parameter than Continuity (Fig S3, right column: from a2 to d2) to represent the effect of vowel context (front - triangles vs. back - rounds) on the experimental round scores. Conversely, Continuity represents better than Balance the gradual increase in round score from unvoiced (empty red shapes) to voiced stops (filled red shapes) to sonorants (filled blue shapes). Continuity also better represents round score increase from stops (red shapes) to fricatives (green shapes). Other Balance and Continuity variations for each phonetic class between experiments are probably due to differences in acoustic content (e.g., differences in speakers, recording conditions, see Table 2 for more details).

<Figure S3>

The study^2^ leads to the lowest amount of explained variance by the Balance-only model (*r*^2^ = 26% for a boundary *b* around channels 24-25). Due to the monosyllabic structure of the stimuli, predictions did not involve the Continuity parameter. The reduced amount of explained variance probably results from the fact that this study is not a very sensitive measure of the bouba-kiki effect. Indeed, while the experimental rounding score can theoretically vary between 1 and 5, its actual range, when averaged across participants, is restricted to between 2.4 and 3.6, leaving little variance to be explained (Fig. 2).

The last study^13^, involved synthetic oral vowels varying in place (front vs. back) and height (high vs. low). Due to the Vowel-only structure of these stimuli, predictions did not involve the Continuity parameter. The best prediction (*r^2^* = 44%) is obtained with a boundary at channel 30 (i.e., 1800 Hz) showing a contrast between stimuli with a high vs. low second formant F2.

***More details on Kac’s paper***^17^ ***about “can we hear the shape of a drum?”***

In this paper in 1966, Kac asks the following question (pp. 2-3):

*“Let Ω_1_ and Ω_2_ be two plane regions bounded by curves Γ_1_ and Γ_2_ respectively and assume that (these two surfaces have exactly the same normal resonance modes). Question: Are the regions Ω_1_ and Ω_2_ congruent in the sense of Euclidean geometry?”* and then he adds, for clarification, *“when I mentioned it to Professor Bers, he said, almost at once: ‘You mean, if you had perfect pitch could you find the shape of a drum’?”*

Then Kac notes (p. 3):

*“I believe that one cannot "hear" the shape of a tambourine but I may well be wrong and I am not prepared to bet large sums either way. What I propose to do is to see how much about the shape can be inferred from the knowledge of all the eigenvalues*” (that is, the normal resonance modes).

Indeed, it will later be demonstrated that the general statement is wrong, since two different surfaces can be constructed with exactly the same resonance spectrum^18^. Still, Kac^17^ shows in his paper that some properties of a surface can be inferred from its resonance spectrum. First, Kac shows that “*one can ‘hear’ the area of Ω*” He proves that the number of mode frequencies lower than a given frequency F is proportional to the area of the surface, hence the larger the area, the larger the number of modes and the lower each mode. This provides the mathematical basis for a classical and well-known fact: larger “drums” are associated with lower resonance frequencies.

From this,^17^ adds an additional finding providing a first solution to the bouba-kiki enigma. Indeed, when refining his mathematical analysis, Kac shows that the distribution of the resonance modes also depends on an additional second-order correcting factor driven by the perimeter of the surface boundary. The smaller the perimeter for a similar surface size, the lower the mode frequencies. Crucially, it is well-known that, for a given surface area, the shape minimizing the perimeter is a circle. This provides a direct link between spectral balance and perceptual roundness. Indeed, the rounder a shape, the smaller its perimeter and the lower its mode frequencies. Hence, the rounder a shape the lower its spectral balance. Conversely, increasing spikiness of a drum increases its perimeter and hence makes its spectrum expand towards higher frequencies.

***Further discussion on the Beating Toys and the Rolling Balls Experiments***

Of course, the Beating Toys and the Rolling Balls Experiments capitalize on very simple and basic scenarii and experimental materials, that call for more experimental manipulations in the future. Indeed, the stimuli in the Beating Toys Experiment would certainly vary to a certain extent depending on the objects texture (wood or metal objects would lead to quite different spectra) and also on the beating dynamics (softer or harder). The stimuli in the Rolling Balls Experiment have shapes maximizing the distinction between round and spiky objects, and less extreme shapes could decrease the corresponding spectral and possibly dynamic contrast (see a discussion on the local vs. global nature of roundness vs. spikiness in the Discussion). Moreover, dynamic parameters such as the texture of objects and rolling surface, the surface slope angle and the weight of the objects should also intervene. Additionally, more studies using implicit association tasks, Likert scales instead of binary forced choices and measuring brain's responses to it should be done in order to further investigate how such cross-modal correspondences are processed and influence how we perceive our environment.

Still, the crucial point here is that for a given set of experimental parameters the round vs. spiky contrast clearly emerges in both experiments, both objectively (see Fig. 4b and 5b) and perceptually (see Fig. 4c and 5c), in relation with the two key parameters that are spectral balance and temporal continuity. Exploring all the possible variations around this major contrast is beyond the scope of the present study, but could be of interest for further developments.

.

**Supplementary Information Figures**

**Figure S1.** Global Balance x Continuity model performances for each of the 10 experiments of the meta-analysis and for the Noise Band Experiment**.** a. Variations of explained variance *r*^2^ as a function of the value of the boundary *b.* It is varied between channel 20 (800 Hz) and channel 30 (1,800 Hz). b. Variance *r*^2^ explained by Continuity-only as a function of *r*^2^ explained by Balance-only for the *b*-value providing an optimal fit between 20 and 30 in a for the nine experiments for which both Balance and Continuity can be computed.

**Figure S2**. Experimental round score (on the same scale as in the original studies) as a function of Balance (a1 & b1) or Continuity (a2 & b2) in both Exp. 1 of the two studies^3,9^. Experimental round scores on the y axes and Balance or Continuity values on the x axes are averaged across specific phonetic classes involved in the corresponding experiment. For one study^3^, the bilabial stops /b p/ are in brown, coronal stops /d t/ are in yellow and velar stops /g k/ are in grey. For the other study^9^, the consonant class grouping is done using the second consonant of the C_1_VC_2_V disyllabic stimuli. Unvoiced and Voiced Stops (US and VS) are in red; Unvoiced and Voiced Fricatives (UF and VF) are in green; Sonorants (S: liquids, glides, nasals) are in blue. For both studies, front vowel context (-fv) are triangles; back vowel context (-bv) are round shapes. Unvoiced consonants are empty shapes, Voiced consonants are filled shapes. Big shapes are averaged values across phonetic classes, while small shapes represent individual speech stimuli values.

**Figure S3.** Experimental round score (on the same scale as in the original studies) as a function of Balance (left column) or Continuity (right column) in four studies represented in a1&2^4^, b1&2^1^, c1&2^6^ and d1&2^5^. For all studies, unvoiced and voiced stops are in red; unvoiced and voiced fricatives are in green; sonorants (liquids, glides, nasals) are in blue; front vowel context are triangles; back vowel context are round shapes. Unvoiced consonants are empty shapes, Voiced consonants are filled shapes. Big shapes are averaged values across phonetic classes, while small shapes represent individual speech stimuli values.

**Supplementary Information References**

1. Bottini, R., Barilari, M. & Collignon, O. Sound symbolism in sighted and blind. The role of vision and orthography in sound-shape correspondences. *Cognition* **185**, (2019).

2. Knoeferle, K., Li, J., Maggioni, E. & Spence, C. What drives sound symbolism? Different acoustic cues underlie sound-size and sound-shape mappings. *Sci. Rep.* (2017). doi:10.1038/s41598-017-05965-y

3. D’Onofrio, A. Phonetic Detail and Dimensionality in Sound-shape Correspondences: Refining the Bouba-Kiki Paradigm. *Lang. Speech* **57**, 367–393 (2013).

4. Ahlner, F. & Zlatev, J. Cross-modal iconicity: A cognitive semiotic approach to sound symbolism. *Sign Syst. Stud.* **38**, (2010).

5. Lacey, S. *et al.* Stimulus Parameters Underlying Sound-Symbolic Mapping of Auditory Pseudowords to Visual Shapes. *Cogn. Sci.* **44**, (2020).

6. Fort, M., Martin, A. & Peperkamp, S. Consonants are More Important than Vowels in the Bouba-kiki Effect. *Lang. Speech* **58**, 247–266 (2015).

7. Maurer, D., Pathman, T. & Mondloch, C. J. Maurer, Pathman, Mondloch - 2006 - The shape of boubas Sound–shape correspondences in toddlers and adults.pdf. **3**, 316–322 (2006).

8. Monaghan, P., Mattock, K. & Walker, P. The role of sound symbolism in language learning. *J. Exp. Psychol. Learn. Mem. Cogn.* **38**, 1152–1164 (2012).

9. Peiffer-Smadja, N. & Cohen, L. The cerebral bases of the bouba-kiki effect. *Neuroimage* **186**, (2019).

10. Sidhu, D. M. & Pexman, P. M. Five mechanisms of sound symbolic association. *Psychonomic Bulletin and Review* **25**, (2018).

11. Walker, P. Cross-sensory correspondences and cross talk between dimensions of connotative meaning: visual angularity is hard, high-pitched, and bright. *Atten. Percept. Psychophys.* **74**, 1792–809 (2012).

12. Westbury, C., Hollis, G., Sidhu, D. M. & Pexman, P. M. Weighing up the evidence for sound symbolism: Distributional properties predict cue strength. *J. Mem. Lang.* **99**, (2018).

13. Kwak, Y., Nam, H., Kim, H. W. & Kim, C. Y. Cross-Modal Correspondence between Speech Sound and Visual Shape Influencing Perceptual Representation of Shape: The Role of Articulation and Pitch. *Multisensory Research* **33**, (2020).

14. Fant, G. *Acoustic theory of speech production.* (Mouton and Co, 1960).

15. Stevens, K. N. Acoustic correlates of some phonetic categories. *J. Acoust. Soc. Am.* **68**, (1980).

16. Stevens, K. N. *Acoustic phonetics*. (MIT Press, 1998).

17. Kac, M. Can One Hear the Shape of a Drum? *Am. Math. Mon.* **73**, (1966).

18. Gordon, C., Webb, D. L. & Wolpert, S. One cannot hear the shape of a drum. *Bulletin of the American Mathematical Society* **27**, (1992).

Supplementary Information Dataset

Not all the authors of the experiments included in the meta-analysis shared their data publicly (cf. Table 2). Summary of available data, stimuli and script of the model, as well as the data, stimuli and scripts used in the Noise Band, Beating Toys and Rolling Balls Experiments are available in this osf: <https://osf.io/v9xyd/?view_only=2d207fc228684f1d8ac96ac97f57759c>.
